# Avian Influenza H5N1 Transmission in Households, Indonesia

**DOI:** 10.1371/journal.pone.0029971

**Published:** 2012-01-04

**Authors:** Tjandra Y. Aditama, Gina Samaan, Rita Kusriastuti, Ondri Dwi Sampurno, Wilfried Purba, Misriyah, Hari Santoso, Arie Bratasena, Anas Maruf, Elvieda Sariwati, Vivi Setiawaty, Kathryn Glass, Kamalini Lokuge, Paul M. Kelly, I. Nyoman Kandun

**Affiliations:** 1 Directorate-General Disease Control and Environmental Health, Ministry of Health, Salemba, Jakarta, Indonesia; 2 National Centre for Epidemiology and Population Health, The Australian National University, Canberra, Australian Capital Territory, Australia; 3 National Institute of Health Research and Development, Ministry of Health, Salemba, Jakarta, Indonesia; 4 Population Health Division, Australian Capital Territory Government Health Directorate, Canberra, Australian Capital Territory, Australia; University of Hong Kong, Hong Kong

## Abstract

**Background:**

Disease transmission patterns are needed to inform public health interventions, but remain largely unknown for avian influenza H5N1 virus infections. A recent study on the 139 outbreaks detected in Indonesia between 2005 and 2009 found that the type of exposure to sources of H5N1 virus for both the index case and their household members impacted the risk of additional cases in the household. This study describes the disease transmission patterns in those outbreak households.

**Methodology/Principal Findings:**

We compared cases (n = 177) and contacts (n = 496) in the 113 sporadic and 26 cluster outbreaks detected between July 2005 and July 2009 to estimate attack rates and disease intervals. We used final size household models to fit transmission parameters to data on household size, cases and blood-related household contacts to assess the relative contribution of zoonotic and human-to-human transmission of the virus, as well as the reproduction number for human virus transmission. The overall household attack rate was 18.3% and secondary attack rate was 5.5%. Secondary attack rate remained stable as household size increased. The mean interval between onset of subsequent cases in outbreaks was 5.6 days. The transmission model found that human transmission was very rare, with a reproduction number between 0.1 and 0.25, and the upper confidence bounds below 0.4. Transmission model fit was best when the denominator population was restricted to blood-related household contacts of index cases.

**Conclusions/Significance:**

The study only found strong support for human transmission of the virus when a single large cluster was included in the transmission model. The reproduction number was well below the threshold for sustained transmission. This study provides baseline information on the transmission dynamics for the current zoonotic virus and can be used to detect and define signatures of a virus with increasing capacity for human-to-human transmission.

## Introduction

The avian influenza (AI) H5N1 virus remains of international public health concern due to its pandemic potential. Based on analyses of AI H5N1 outbreaks during 2003 to 2009, most cases were sporadic and had documented exposure to zoonotic sources of the virus [1]. For clusters of AI H5N1 infection, the majority occurred in people who were genetically related to each other and most also had exposure to zoonotic (bird to human) sources of virus [1]. Studies suggest that human transmission of the virus occurred in a very limited way in some clusters [2], [3]. However, the transmission patterns remain largely unknown.

Quantification of transmission patterns such as the probability of both human and zoonotic transmission of the H5N1-virus, the reproduction number (R_0_), secondary attack rates (SAR) and the interval between case onsets are important parameters to inform preparedness and response measures to outbreaks, especially to signal events that indicate changed virus behavior [4]. It is also crucial that both zoonotic and human infection pathways are considered, and results are interpreted in the context of a zoonotic infection with limited transmission among humans [5], [6]. Models that incorporate both the zoonotic and human transmission components are rare [5].

As of July 30, 2009, Indonesia had reported 139 outbreaks of avian influenza (AI) H5N1 infection in humans with a case fatality rate of 85% [7]. The epidemiology of Indonesia's cases has been reported previously [8]–[10]. A recent study on the 139 outbreaks assessed the risk factors for household clustering of cases and the risk factors for who in the household is likely to become a secondary case of H5N1-infection [11]. The study found that the type of exposure to sources of H5N1 for both the index case and their household members impacted the risk of additional cases in the household. The study also added evidence that H5N1 infection may be dependent on host genetic susceptibility since first-degree blood relatives to index cases were at greater risk of becoming secondary cases. However, this study did not assess the attack rates (AR), SAR or transmission parameters in those outbreak households.

To date, only one study has estimated the transmission patterns based on case data in Indonesia [4]. Estimates generated were solely based on one outbreak – a cluster of one probable and seven confirmed cases detected in North Sumatra in 2006. The study found statistical evidence of human-to-human transmission and estimated SAR at 29% and R_0_ at 1.14 [4]. Since data on the total persons exposed and individual factors such as exposure type were not fully available to that study, the transmission pathways were not investigated in detail. Also, since the model was fitted and transmission estimates generated based only on that one cluster, which is considered atypical due to its large size, the estimates are likely to be an over-estimate for outbreaks in Indonesia.

Since Indonesia's cumulative AI H5N1 infection case count represents one-third of the world's cases, the outbreak transmission patterns are of international importance. Building on previous findings about the epidemiology of H5N1 infection in households [11], we describe infection AR, infection SAR, risk factors for H5N1 infection and intervals between case illness onsets. We then estimate transmission parameters and quantify the relative contribution of zoonotic and human transmission as well as the extent to which the virus was transmissible between people (reproduction number). While international data suggest most transmission is zoonotic, there is also some evidence of human-to-human transmission [2], [12]. We fitted household models to the Indonesian data that allow for both zoonotic and human-to-human transmission to assess the extent of transmission from each source and to provide an estimate of the reproduction number in the case that human-to-human transmission occurs.

## Results

A total of 139 outbreaks of human AI H5N1 infection were detected in Indonesia in the four year study period. There were 113 sporadic case outbreaks and 26 cluster outbreaks. The total number of cases was 177, with 64 cases in the 26 clusters. Only one cluster had over four cases; the North Sumatran cluster of 2006, which can be considered an outlier based on its large size of seven confirmed and one probable case. There were 535 household contacts to index cases in the study, of which blood relation was known for 94% (n = 503). Most of the 503 contacts were blood relatives (n = 383, 76%) and 120 (24%) were non-blood relatives. None of the non-blood related household contacts became secondary cases.

### Household Study

For the 80 outbreaks for which household and contact data were available, the proportion of cluster to sporadic outbreaks increased as household size increased (Table 1). To highlight the impact of the outlier cluster on the AR and SAR, findings are presented both including and excluding that cluster. The overall AR was 17.8% (103 cases / 579 exposed) when the outlier cluster was excluded and 18.3% (111 cases / 607 exposed) when included. There was a stable SAR between 3.1–4.5% across household size (Table 1). However, inclusion of the outlier cluster inflated SAR for households with>15 persons to 12.5% (Table 1). These findings are consistent with predominantly zoonotic virus transmission. In the absence of human transmission, and with low levels of zoonotic transmission, the AR would be expected to decline with household size, while the SAR should remain roughly constant.

**Table 1:** Household size and secondary attack rate for outbreaks of avian influenza H5N1 infection.

Contact data	Household size	Outbreak size (confirmed and probable cases)								Total outbreaks	Proportion cluster	Total contacts	Secondary cases	SAR
		1	2	3	4	5	6	7	8					
Available	1–5	27	4	1	0	0	0	0	0	32	0.16	152	9	0.059
	6–10	25	6	2	0	0	0	0	0	35	0.24	219	8	0.036
	11–15	8	3	0	1	0	0	0	0	12	0.33	85	3	0.035
	>15	0	2	0	0	0	0	0	1	3	1.00	69	9	0.130^a^
	Sub-total	60	15	3	1	0	0	0	1	80	0.24	525	29	0.055^b^
Not available		53	5	1	0	0	0	0	0	59	0.10	-	6	-
Total		113	20	4	1	0	0	0	1	139	0.19	-	35	-

a SAR declines to 0.047 when outlier cluster is excluded.

b SAR declines to 0.044 when outlier cluster is excluded.

Cases (nz = 177) and healthy contacts (n = 496) were compared to assess risk factors for infection (Table 2). Young age groups (≤ 30 years) were at increased risk of infection, where individuals between five and 17 years of age had 3.5 times the odds to be infected when compared with those >30 years of age [Adjusted Odds Ratio (aOR) = 3.44, 95% Confidence Interval (CI)) 1.86–6.36]. Most cases (87%) and their healthy contacts (69%) had zoonotic exposure. However, direct exposure to zoonotic sources of AI H5N1 virus tripled the odds of infection (aOR = 3.08, 95% CI 1.54–6.13). Lastly, small households (1–5 persons) were significantly more likely to have cases than households with >5 people (Table 2). The final multivariate model with three variables had good fit (p = 0·17).

**Table 2:** Comparison of cases (n = 177) and healthy contacts (n = 496) in outbreaks of avian influenza H5N1 infection.

			Univariate	Multivariate a	
Variable	Cases, n (%)	Healthy contacts, n (%)	OR(P-value)	Adjusted OR(P-value)	95% CI
Age groups (years) b					
0–4	18 (10)	41 (9)	2.66 (0.004)	3.18 (0.004)	1.45–6.98
5–17	65 (37)	96 (21)	4.11 (<0.001)	3.44 (<0.001)	1.86–6.36
18–30	61 (35)	125 (28)	2.96 (<0.001)	3.20 (<0.001)	1.81–5.68
>30	31 (18)	188 (42)	Reference group	-	-
Sex					
Male	83(47)	225 (47)	0.99 (0.94)		
Female	94 (53)	258 (53)			
Exposure					
Direct zoonotic	81 (46)	130 (26)	4.02 (0.002)	3.08 (0.001)	1.54–6.13
Indirect zoonotic	72 (41)	211 (43)	2.20 (<0.001)	1.43 (0.29)	0.72–2.81
Inconclusive zoonotic	24 (13)	155 (31)	Reference group	-	-
Household size (persons) c					
1–5	51 (46)	143 (29)	Reference group	-	-
6–10	38 (34)	211 (43)	0.51 (0.009)	0.50 (<0.001)	0.34–0.73
11–15	10 (9)	82 (16)	0.35 (0.001)	0.32 (<0.001)	0.18–0.57
>15	12 (11)	60 (12)	0.51 (0.07)	0.40 (0.16)	0.11–1.43

a Observations  = 561, Goodness-of-fit test: P = 0.17, OR denotes odds ratio, CI denotes confidence interval. OR were adjusted for the inclusion of the three variables in the final multivariate model.

b Data missing for two cases and 46 healthy contacts.

c Data missing for 66 cases from the 59 outbreaks for which household data were not available.

**Figure 1:**
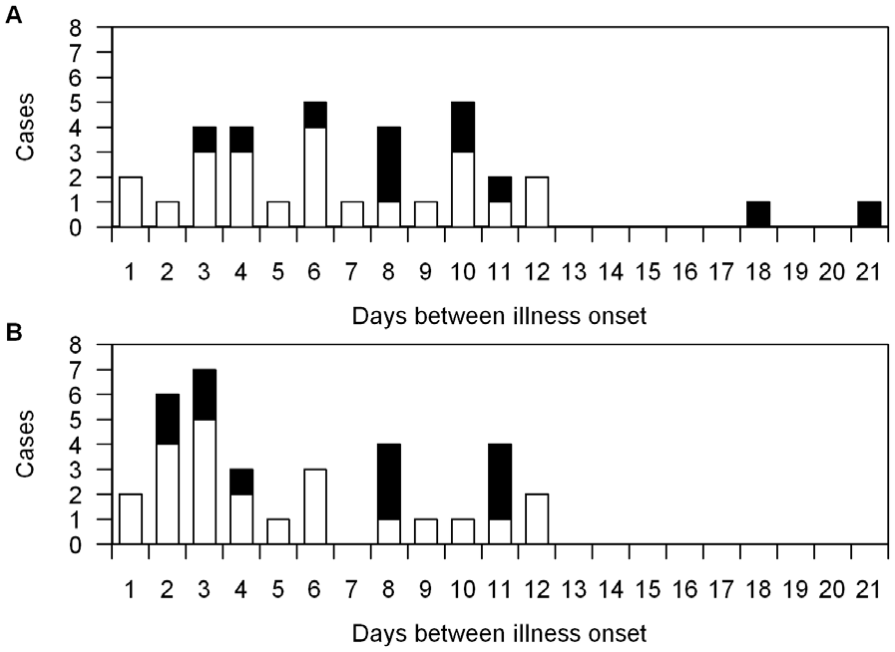
Interval between onset of illness for cases (n = 34) in outbreaks of avian influenza H5N1 infection. Panel A shows the interval between onsets of illness of index and secondary cases in outbreaks. Panel B shows the interval between onsets of illness of serial cases in outbreaks. Black denotes cases not exposed to zoonotic sources of virus and white denotes cases exposed to zoonotic sources of virus.

In cluster outbreaks, the median interval between the index case onset and secondary case onset of illness was 8 days (range 1–21 days, Figure 1A). The median interval between the onset of illness of a secondary case and the previous case in the same outbreak was 6 days (range 1–12 days, Figure 1B). Based on the investigation reports, eleven secondary cases had inconclusive exposure to a zoonotic source of virus. All of these had onset of illness at least two days after the index case's onset of illness. For these 11 cases, the median interval between illness onset of serial cases was 8 days (range 2–11 days, Figure 1B).

### Transmission Model

To assess the exposure of secondary cases, Table 3 presents the transmission analysis comparing three model types: all transmission from zoonotic sources (Model A), all transmission was human transmission (Model B) and transmission was from both zoonotic and human sources (Model C). Two denominator populations are presented for comparison; all exposed individuals in outbreaks and all exposed blood-related household members to index cases. The final column of the tables shows the percentage support for the models, which can be interpreted as the probability that the model is the best among those considered. To highlight the impact of the outlier cluster on transmission parameters and model selection, findings for two datasets are presented; one with the outlier cluster included and one with it excluded.

**Table 3:** Transmission parameters for outbreaks of avian influenza H5N1 infection.

Data	Denominator population	Model description	Mean human transmission cases a (95% CI)	Mean zoonotic infected cases b (95% CI)	AIC_C_ percent support c
80 outbreaks (North Sumatra cluster included)	All exposed individuals	A) Only zoonotic transmission	-	0.276 (0.126, 0.476)	0.1
		B) Only human transmission	0.172 (0.026, 0.322)	-	6.3
		C) Full model	0.115 (0.009, 0.315)	0.094 (0.000, 0.344)	4.4
	All exposed blood-relatives	A) Only zoonotic transmission	-	0.385 (0.185, 0.635)	2.2
		B) Only human transmission	0.231 (0.082, 0.382)	-	42.9
		C) Full model	0.140 (0.004, 0.390)	0.157 (0.000, 0.452)	44.0
79 outbreaks (North Sumatra cluster excluded)	All exposed individuals	A) Only zoonotic transmission	-	0.221 (0.071, 0.421)	5.1
		B) Only human transmission	0.166 (0.024, 0.316)	-	2.4
		C) Full model	0.052 (0.000, 0.302)	0.158 (0.000, 0.403)	2.5
	All exposed blood-relatives	A) Only zoonotic transmission	-	0.310 (0.110, 0.510)	53.3
		B) Only human transmission	0.227 (0.077, 0.427)	-	14.0
		C) Full model	0.052 (0.000, 0.352)	0.242 (0.000, 0.542)	22.7

a Mean number of secondary cases infected by a single index case in an exposed population of size 5, CI denotes confidence interval.

b Mean number of zoonotic cases in an exposed population of size 5.

c AIC_C_ denotes Akaike Information Criterion adjusted for small sample size. This indicates the percent probability that the model is the best amongst those considered.

Regardless of the denominator population or the dataset, there was much less support for Model A (zoonotic transmission only) than either Models B (human transmission only) or C (combination of zoonotic and human transmission) (Table 3). This was confirmed by a simulation-based test of model fit, which demonstrated significant differences between Model A and the data (p<0.01 for both). Despite significant evidence that human transmission occurred when the outlier cluster was included in the analysis, estimated human transmission rates were low with the reproduction number lying between 0.1 and 0.25, and the upper confidence bounds all below 0.4 for an exposed population of five individuals. Estimated zoonotic transmission rates ranged from 0 to 0.38 cases in an exposed population of five household members.

When the analysis excluded the outlier cluster (Table 3), similar estimates for the human transmission parameters and the reproduction number were found, but there was no longer significant evidence of human transmission. Indeed, the model with the strongest support was Model A (zoonotic transmission only), with 0.31 zoonotic cases infected in an exposed population of five household members. This suggests that the main evidence for human transmission comes from the outlier cluster. For all model types, both including and excluding the outlier cluster, use of blood-related household members as the denominator population provided better model fit. A test of the sensitivity of our results to the households in which contact data were missing found very little change to the transmission estimates, with estimates of zoonotic transmission parameters reduced by around 0.05–0.1 cases in an exposed population of size 5, point estimates of human transmission parameters largely unchanged, and a decrease in the upper bound of the human transmission parameter of 0.02–0.08 cases in an exposed population of size 5.

## Discussion

This study is the first globally to examine AI H5N1 transmission patterns in households for a large number of outbreaks aimed at quantifying human-to-human transmission of the AI H5N1 virus. The study had three main findings. Firstly, most cases of AI H5N1 infection were a result of exposure to zoonotic sources of virus. In fact, the study only found strong support for human transmission of the virus when a single large cluster was included in the transmission model. Secondly, the overall SAR was 5.5% in the 80 outbreaks for which household contact data were available. This was much lower than previous estimates [4]. Thirdly, the study adds evidence that blood relatives are at greatest risk of becoming secondary cases in outbreak households. This adds support to the hypothesis that there is an element of genetic susceptibility to AI H5N1 infection [3].

The finding that the AI H5N1 virus does not transmit efficiently between humans and that infection remains primarily zoonotic impacts the interpretation of the interval between case onsets and the SAR. These parameters should not be interpreted as human-to-human transmission parameters. Rather, the interval between case onsets (median 6 days, range 1–12 days) represents observed timelines between human cases during an epizootic and indicates the duration of risk of more cases being detected in association with the epizootic event. This information can guide the length of contact tracing needed to detect and prevent further cases during an outbreak. The findings from this study reinforce the WHO recommendation to trace and monitor case contacts for two weeks after the illness onset of the last case [13].

The SAR results add to the body of knowledge on typical outbreak size associated with the current zoonotic virus, where SAR remained approximately stable with household size. This provides important baseline information for future outbreak investigations and may help in the detection of changes in virus behavior. For a virus on the verge of efficient human spread, the household SAR should be compared to the current findings as well as SAR for other influenza viruses.

Although the SAR remained stable with household size, the proportion of outbreaks with more than one case increased with household size. This highlights an important distinction between individual and household risk for infection with the current zoonotic virus: a person in a large household is less likely to be infected than a person in a small household, but large households are more likely to have a secondary case than small households. Whether SAR was low due to virus and host characteristics or due to public health interventions such as prophylaxis of case contacts or isolation of cases was not explored in this study, but warrants future investigation. Importantly, the SAR could not be calculated for the remaining 59 outbreaks as contact data were not available to determine the household size. The missing data highlight the challenge in standardizing data collection for a new emerging disease. However, as the excluded outbreaks were typically smaller than those with full contact data (90% of excluded outbreaks were sporadic), it seems unlikely that inclusion of those outbreaks would increase the overall SAR or the transmission parameters. Our sensitivity analysis suggested that inclusion of these data would likely result in a slight decrease in the zoonotic transmission parameter, negligible impact on the point estimate of the human transmission parameter, and a slight decrease in the upper bound of the human transmission parameter.

Due to the limited sensitivity of public health surveillance systems, varied health-seeking behavior within the population and the potential for mild infections, it is possible that cases or clusters of H5N1 infection were missed and not included in the analysis. This affects our findings. If sporadic cases of H5N1 infection resulting from zoonotic transmission of the virus were missed, then our study likely over-estimates overall SAR and transmission parameters. If clusters of cases were missed, then our study may under-estimate these parameters. We speculate, based on our H5N1 case investigations, that clusters of disease are less likely to be missed than sporadic cases of infection since families and healthcare workers would raise alarms in the public health system about multiple cases of pneumonia in a single household. For mild cases, it is feasible that cases are missed, which suggests that our results would under-estimate transmission parameters. However, based on studies conducted amongst poultry workers exposed to H5N1 virus in the course of their work, mild and subclinical infections have been limited [14]–[16]. This is also mirrored in influenza virological surveillance findings conducted by countries affected by H5N1 virus such as Lao PDR, China and Cambodia, whereby these sentinel surveillance systems regularly detect seasonal influenza viruses circulating in the community and in hospital settings, yet they rarely detect cases of H5N1 virus infection [17]–[19].

The disease transmission model achieved a better fit when the exposed population was restricted to blood-related household members. The study also found that only blood relatives to the index case developed illness and that none of the 120 non-blood related household members (such as spouses and family-in-law) developed illness. Collectively, these findings add evidence to the hypothesis that there is a host genetic effect on susceptibility to AI H5N1 infection [11]. However, since genetic relationship and household membership are correlated, it is difficult to identify the mechanisms most responsible for household clustering. Thus, further research is needed to explore these findings.

Individuals at most risk of infection were those ≤30 years, especially children between five and 17 years. The young age pattern was also observed globally based on analysis of cases from 11 countries [1]. This suggests that young age groups have greater susceptibility to AI H5N1 infection; be it due to social, hygienic or biological factors. Potential reasons include that children are more likely to handle sick and infected birds or to be exposed to contaminated environment through play or through bird rearing. In Indonesia, anecdotal evidence suggests that bird rearing is delegated to young household members. Children are less conscious of hygiene and thus may have had unprotected interaction with sources of virus [20].

Household based studies exploring risk factors for infection are less likely to be affected by case-ascertainment bias [21]. However, since household data were not available for all outbreaks, our analyses and conclusions were based on a restricted dataset and should be interpreted with caution as the missing data limit the power of our study. Nevertheless, as discussed earlier, since 90% outbreaks lacking household data only had one case, our study likely over-estimated the transmission parameters and the SAR, indicating that human transmission rates were very low.

Overall, the study found that AI H5N1 human infection resulting from human transmission of the virus was very limited, and that the reproduction number was well below the threshold for sustained transmission. Case clustering does not always denote human transmission of the virus, but is often the result of household members' shared exposure to zoonotic sources of the virus [22]. The study findings also suggest that there may be a host genetic effect on susceptibility to infection, but this warrants further investigation through epidemiological and immunological studies to untangle the correlation between household membership, shared exposures and genetics.

## Materials and Methods

### Ethics Statement

All data in this study were obtained from the case-investigation reports and the surveillance database at the Ministry of Health, which were collected as part of an ongoing public-health investigation. Permission to conduct the study and analyze the data was obtained from the data custodian (first author, Director-General for Disease Control and Environmental Health at the Ministry of Health, Republic of Indonesia). Data shared with international study collaborators, who were not involved in the case investigations, were de-identified to protect the confidentiality of the cases and their families, whereby names and addresses were removed. Ethics approval for the study was obtained from the Australian National University's Human Research Ethics Committee.

### Setting

The Ministry of Health AI H5N1 case database and detailed case investigation forms were reviewed and analyzed for cases detected in Indonesia between July 2005 and July 2009. The study conformed with the WHO definitions [13], whereby a cluster is a group composed of one confirmed case of H5N1 virus infection and additional confirmed or probable cases associated with a specific setting, with the onset of cases occurring within 2 weeks of each other. In households with a cluster of cases, the index case was defined as the one with the earliest symptom onset date amongst all the cases in that household. A sporadic outbreak was defined as one confirmed case of H5N1 virus infection. Case definitions for probable and confirmed cases were based on the WHO definitions described previously [23]. For both sporadic and cluster outbreaks, a household contact was a person who had at least four hours contact with a probable or confirmed case at home within the seven days prior or 14 days after the case's onset of illness.

### Data Collection

Field investigation teams investigated every outbreak. Teams interviewed cases when possible (since many cases died before investigation teams arrived), family members and key informants such as healthcare workers. As described previously [8], [11], data were collected using a standardized H5N1-case questionnaire developed by the Ministry of Health based on WHO guidance [24]. The questionnaire collected data on the case's household, clinical symptoms, healthcare facility attendance and potential zoonotic, human and environmental exposures to sources of H5N1-virus. Medical records from all healthcare facilities visited by cases during the course of their illness were reviewed and extracted to complete the questionnaire.

Contact tracing, clinical examination and testing of household contacts were done during the investigation. Serum samples were collected from all healthy household contacts to assess for H5N1 seroconversion using microneutralization test or haemagglutination inhibition test (with horse red blood cells). For household contacts with symptoms of H5N1 infection, nasal and throat swabs were collected and tested using real-time reverse transcriptase polymerase chain reaction (RT-PCR) test. All tests were conducted according to the WHO guideline on recommended laboratory procedures for H5N1 detection [25]. Healthcare workers from the nearest government primary healthcare centre were instructed to visit the household daily for two weeks to monitor and detect any additional cases.

### Household Study

AR, SAR, risk factors for infection and intervals between case onsets were analyzed in a household-based study. Household size was the number of people in the household including cases. A household contact was a person who had at least four hours contact with a case at home within the seven days prior or fourteen days after a case's onset of illness. AR was calculated for the 80 outbreaks (60 sporadic and 20 clusters) out of the 139 for which household data were available. Data on household contacts were missing for 59 outbreaks, of which 90% (n = 53) were sporadic case outbreaks and the largest outbreak involved three cases. AR was defined as the proportion of people who met the definition for confirmed or probable AI H5N1 infection in the outbreak (household). SAR was defined as the proportion of household contacts who met the probable or confirmed case definition after the onset date for the index case and within two weeks of the onset of symptoms of a prior household case. Two weeks was selected as the maximum follow up period based on WHO guidance [13]. The intervals (days) between onset of symptoms of index cases and subsequent cases in clusters, and the interval between serial cases in clusters were calculated.

Logistic regression models that accounted for household clustering using a cluster robust standard error for the coefficients were used to evaluate the risk factors for infection. Multivariate models were constructed using variables significant at p = 0.1 in the univariate analyses. A final model was achieved by sequentially discarding terms not significant at P = 0.05 starting with the ones with the highest P-values. We used the le Cessie-van Houwelingen-Copas-Hosmer unweighted sum of squares goodness-of-fit test to assess model validity, as advocated by Hosmer *et al.* 26–28. Stata software version 10.0 (StataCorp) was used for this analysis.

Four variables were explored as risk factors for infection: age, sex, exposure type and household size. To simplify interpretation of results, age and household size were analyzed categorically. Categories were based on data spread; four groups for age in years (0–4, 5–17, 18–30 and ≥31) and four groups for household size (1–5, 6–10, 11–15 and >15 people). Exposure was defined as whether the individual had direct, indirect or inconclusive zoonotic exposure to a source of AI H5N1 virus. Direct zoonotic exposure referred to cases who handled sick or dead poultry, handled poultry products such as fertilizers, or who had poultry deaths in the home. Indirect zoonotic exposure referred to cases where poultry deaths were reported in the neighborhood, cases where healthy poultry were present in the neighborhood and cases who visited live bird markets. Inconclusive zoonotic exposure refers to cases where no zoonotic source of infection could be found despite investigation.

### Transmission Model

To assess the potential for human transmission of the virus, we used final size household models to fit the human and zoonotic transmission parameters to outbreak data (household size, number of cases, blood-related household members to index case) in a manner similar to that described in van Boven *et al.* [29]. This approach allows for both human and zoonotic transmission, and enables comparison of different transmission assumptions. We used Akaike Information Criterion adjusted for small sample size (AIC_C_) to select the most appropriate models. The AIC_C_ percent support gives the probability that the model is the best model of those considered, but does not indicate how well the suite of models fit the data [30]. We used a simulation-based approach to compare the data with each of the model predictions, which allowed us to identify those models that differed significantly (P<0.05) from the data. Matlab (version R2010b) was used for this analysis. Our preliminary analysis indicated that density-dependent transmission [31] gave a better fit to the data than frequency-dependent transmission [29], and that assumptions concerning the distribution of the infectious period did not affect our results. Thus, our detailed analysis used a model with a fixed infectious period and density-dependent transmission. Under these assumptions, outbreak sizes will vary according to the exposed population, and we present results for an exposed population of size five (the median household size in the data).

Our estimation methods calculated the best-fit parameters to cluster data consisting of the number of exposed individuals, the number of index cases and the final outbreak size. In our initial analysis, we used all individuals exposed for a period of four hours or more in the household as the exposed population. In light of evidence concerning transmission of H5N1 to blood-related contacts [1], [3], we also considered an alternative analysis in which the exposed population was restricted to all blood relatives exposed for a period of four hours or more in the household. Finally, we tested the sensitivity of our results to the inclusion of those households for which contact data were missing, by including the missing households into the data, assuming that they had 5 household members (the median household size in the data) and 4 blood relative contacts (again, the median in the data).
